# De novo metatranscriptome assembly and coral gene expression profile of *Montipora capitata* with growth anomaly

**DOI:** 10.1186/s12864-017-4090-y

**Published:** 2017-09-11

**Authors:** Monika Frazier, Martin Helmkampf, M. Renee Bellinger, Scott M. Geib, Misaki Takabayashi

**Affiliations:** 1 Tropical Conservation Biology and Environmental Science, University of Hawaiʻi at Hilo, 200 West Kāwili Street, Hilo, HI 96720 USA; 2 United States Department of Agriculture, Agriculture Research Service, Daniel K Inouye U.S. Pacific Basin Agricultural Research Center, 64 Nowelo St, Hilo, HI 96720 USA; 3 Marine Science Department, University of Hawaiʻi at Hilo, 200 West Kāwili Street, Hilo, HI 96720 USA

**Keywords:** Coral disease, Gene expression, Growth anomaly, RNA-seq, Metatranscriptome, *Montipora capitata*

## Abstract

**Background:**

Scleractinian corals are a vital component of coral reef ecosystems, and of significant cultural and economic value worldwide. As anthropogenic and natural stressors are contributing to a global decline of coral reefs, understanding coral health is critical to help preserve these ecosystems. Growth anomaly (GA) is a coral disease that has significant negative impacts on coral biology, yet our understanding of its etiology and pathology is lacking. In this study we used RNA-seq along with de novo metatranscriptome assembly and homology assignment to identify coral genes that are expressed in three distinct coral tissue types: tissue from healthy corals (“healthy”), GA lesion tissue from diseased corals (“GA-affected”) and apparently healthy tissue from diseased corals (“GA-unaffected”). We conducted pairwise comparisons of gene expression among these three tissue types to identify genes and pathways that help us to unravel the molecular pathology of this coral disease.

**Results:**

The quality-filtered de novo-assembled metatranscriptome contained 76,063 genes, of which 13,643 were identified as putative coral genes. Overall gene expression profiles of coral genes revealed high similarity between healthy tissue samples, in contrast to high variance among diseased samples. This indicates GA has a variety of genetic effects at the colony level, including on seemingly healthy (GA-unaffected) tissue. A total of 105 unique coral genes were found differentially expressed among tissue types. Pairwise comparisons revealed the greatest number of differentially expressed genes between healthy and GA-affected tissue (93 genes), followed by healthy and GA-unaffected tissue (33 genes), and GA-affected and -unaffected tissue (7 genes). The putative function of these genes suggests GA is associated with changes in the activity of genes involved in developmental processes and activation of the immune system.

**Conclusion:**

This is one of the first transcriptome-level studies to investigate coral GA, and the first metatranscriptome assembly for the *M. capitata* holobiont*.* The gene expression data, metatranscriptome assembly and methodology developed through this study represent a significant addition to the molecular information available to further our understanding of this coral disease.

**Electronic supplementary material:**

The online version of this article (10.1186/s12864-017-4090-y) contains supplementary material, which is available to authorized users.

## Background

### Coral health

As the foundational organisms of coral reefs, scleractinian corals play a vital role in the health of these diverse ecosystems [1]. The coral holobiont (i.e. the coral host along with endosymbiotic dinoflagellates, *Symbiodinium* spp., and other eukaryotic and prokaryotic symbionts) provides a high rate of primary production, which supports the high biodiversity, functional complexity and productivity found in coral reefs, despite the oligotrophic nature of the tropical waters which they inhabit. Natural and anthropogenic factors have lead to the rapid decline of coral reef ecosystems across the globe in recent decades. Coral reefs are especially vulnerable to loss in low coral diversity regions such as Hawaiʻi [2]. Coral diseases have been recorded in over 100 species around the globe [3], and are a major threat to coral and coral reef ecosystem health [4]. Coral diseases are thought to arise from influences of abiotic and biotic factors, and have been suggested as biological indicators of disturbance and stress on coral reefs [3]. In some cases, coral disease severity has been linked to anthropogenic factors [5, 6]. Since the prevalence of coral diseases are predicted to increase as a consequence of global climate change [7], the effective management of coral reefs must incorporate knowledge of the potential causes and effects of coral diseases [8].

### Coral growth anomaly

Growth anomaly (GA; also referred to as skeletal growth anomaly or skeletal tissue anomaly in previous literature) is a widespread coral disease—one of only four coral diseases that have been identified at multiple locations around the world [9, 10]. GA has been documented in 40 species of scleractinian corals from 20 genera in the Indo-Pacific and Caribbean [9, 10]. To date, studies of coral GA have focused on the morphology, histopathology, ecology, and the physiological effects of GA on the coral host, with the exception of a recent study in which researchers utilized RNA-seq to assess differential gene expression between healthy and GA-affected tissue in the coral *Platygyra carnosa* [11]. GA is characterized by circumscribed lesions with abnormal skeletal and tissue structure, including reduced density of polyps and symbiotic algae, although GA morphology varies among species [12–18]. Reduced density of coral polyps and symbiotic algae can result in decreased colony fitness, as polyps capture food sources from the water column and symbiotic algae produce energy used for coral growth and reproduction [19–21]. Further reduction of photosynthetic capacity has been measured in *Montipora capitata* GA via quantum yield, suggesting that the micromorphology of GA lesions leads to high light stress, causing photoinhibition [22]. Decreased reproductive capacity in GA-affected corals, evidenced by decreased density and partial development of gonads in GA tissue, has been observed in corals of the genera *Acropora* [14], *Montipora* [16], and *Porites* [13].

Histopathological analyses of GA have revealed hyperplasia (tissue enlargement caused by increased cell production) of the tissue connecting polyps, possibly due to the increased need for energy transport from adjacent healthy to diseased tissue [13, 16]. This transport of energy results in decreased growth rate of the adjacent, apparently healthy, tissue compared to tissue from healthy coral colonies, as well as increased GA growth as connectivity to adjacent tissue increases [12, 13, 20]. Though coral GA exerts a significant decrease in fitness of GA-affected colonies, it generally does not result in colony mortality [13, 20].

Our current understanding of the pathology of GA is incomplete, and mostly based on small sample sizes, short-term assessments, and contradicting evidence for GA pathology among studies. Potential predictors of GA prevalence include environmental factors such as coastal development [23], poor water quality [24], human population density [6, 25], coral host density [6] and high sea surface temperatures associated with coral bleaching [14, 26], although lack of a clear etiology limits our understanding of the effects of biotic and abiotic factors on GA prevalence and severity. Studies have identified significantly higher GA prevalence in the central (oldest) region of coral colonies as well as in larger colonies [13–15], leading some to suggest that GA is the result of natural senescence of corals [14].

Since the main sign of this disease is the anomalously enlarged skeletal growth, some studies have taken oncological research approaches to coral GA. Histological and molecular studies targeting specific oncogenes and proteins have presented inconclusive and conflicting evidence for GA being hyperplastic in both *Porites compressa* [13] and *M. capitata* [16] and neoplastic (uncontrolled growth of cells that is not under physiologic control) in *M. capitata* [27]. A more recent study broadened the focus beyond oncogenes to a meta-transcriptomic analysis of the coral *P. carnosa* to identify genes affected by GA, yielding new insights into the impact of GA on osteogenesis, oncogenesis, and the immune system [11]. The purpose of the present study was to employ a similar meta-transcriptomic approach to elucidate the molecular processes of the coral *M. capitata* affected by GA. Specifically, we compared transcriptome profiles of coral tissues sampled from healthy colonies (“healthy”), unaffected tissues sampled from GA-affected colonies (“GA-unaffected”), and tissues sampled directly from GA lesions (“GA-affected”).

## Results and discussion

### Coral holobiont metatranscriptome

We assembled 687 million RNA-seq reads into a metatranscriptome based on 27 *M. capitata* tissue samples, resulting in 87,085 transcripts (76,063 genes) after quality filtering (Table 1). Since the composition and genetic activity of the diverse community of organisms harbored by corals may change with tissue type and disease status, we conducted multi-dimensional scaling (MDS) and differential gene expression (DGE) analyses to study how GA affects gene expression at the level of the holobiont. MDS ordination of gene expression profiles revealed no consistent similarity within or between healthy, GA-affected, or GA-unaffected tissue samples (Additional file 1: Figure S1). In addition, pairwise comparisons of holobiont gene expression among tissue types showed only three significant differentially expressed genes (DEGs) between healthy and GA-affected tissue types (False Discovery Rate (FDR)-adjusted *p*-value ≤0.0009). Of the two genes that were upregulated in healthy as compared to GA-affected tissue, one encodes an uncharacterized protein, and the other is a putative transposase of the IS4 family, which presumably catalyzes the excision and insertion of transposable elements. The gene that was upregulated in GA-affected as compared to healthy tissue is uncharacterized, but contains a PB1 domain, which is frequently found in cytoplasmic signaling proteins in eukaryotes. The lack of a clear holobiont-level gene expression profile of GA, and the unusually low number of DEGs among such distinct tissue types may be an indication of the complexity of the holobiont metatranscriptome and its response to GA.

**Table 1:** Descriptive statistics for *M. capitata* de novo*-*assembled metatranscriptome, quality filtering criteria and putative coral transcriptome

	Genes^a^	Isoforms^b^	%GC^c^	Total bp^d^	Mean bp^e^	N50 bp^f^
De novo assembly	441,520	660,340	45.5	601,736,076	911	1556
FPKM ≥0.5^g^	146,298	237,332	46.5	307,002,357	1293	1916
Complete ORF^h^	46,876	91,876	46.4	209,031,715	2275	2689
Internal ORF	23,610	27,492	53.7	24,431,475	888	1177
5′ Partial ORF	53,546	76,197	52.4	124,301,357	1631	1929
3′ Partial ORF	14,368	20,431	49.3	31,264,758	1530	1912
<90% Similarity^i^	114,925	137,299	50.8	214,880,995	1565	1956
QF assembly^j^	76,063	87,085	50.6	143,828,498	1652	1996
Coral transcriptome^k^	13,643	20,461	41.6	39,739,502	1942	2409

^a^Genes refers to Trinity-assembled contigs. ^b^Isoforms refers to Trinity-assembled isotigs. ^c^%GC is the percent of nucleotide bases in sequences that are either G or C.^d^Total bp is the total number of basepairs in the given assembly or subset thereof. ^e^Mean bp is the average length of assembled contig. ^f^ N50 bp is the mean number of basepairs in all transcripts that, ordered by length, make up 50% of the assembly. ^g^FPKM = fragments per kilobase of transcript per million mapped reads; sum of pooled samples ≥0.5. ^h^ORF = open reading frame; sequences containing a complete, internal or partial ORF were included in the quality-filtered metatranscriptome assembly. ^i^protein sequences with <90% similarity (for proteins with >90% similarity to each other, the longest sequence was retained as the representative sequence for that cluster). ^j^Quality-filtered metatranscriptome assembly. ^k^Putative coral transcriptome (see Methods for coral transcript identification criteria)

### Coral transcriptome

**Fig. 1:**
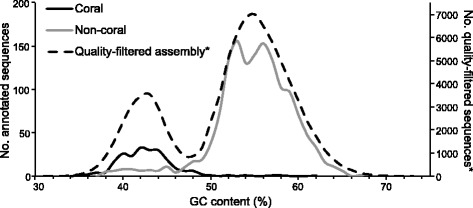
GC content of quality-filtered assembly transcripts by taxon. Comparing the GC content distribution of transcripts in the quality-filtered metatranscriptome with putative homology to higher level holobiont taxa shows that coral host transcripts are characterized by a lower GC content. A GC% content cutoff was used to classify transcripts that were not annotated to any coral holobiont taxa in an effort to include potentially novel coral transcripts in our gene expression analyses. Note that the y-axis values for the quality-filtered assembly (dashed line) are displayed on the secondary (right) axis

**Fig. 2:**
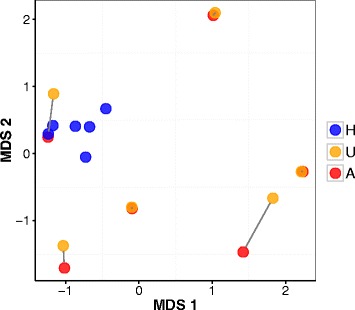
Coral host gene expression profiles of healthy (H), GA-affected (A) and GA-unaffected (U) tissue samples. In this metric MDS (multidimenisonal scaling) plot, distances between each pair of samples represent the typical log_2_ fold-change in gene expression between transcripts. Healthy samples are more similar to each other than to GA-affected or GA-unaffected samples. Conversely, GA-affected and GA-unaffected samples are much more variable and group by colony instead of GA status (gray lines connect GA-affected and GA-unaffected samples derived from the same colony)

Using homology to annotated proteins along with taxon-specific biases in relative GC-content (Fig. 1), we partitioned the metatranscriptome into coral host and symbiont subsets. We identified 20,461 transcripts representing 13,643 genes that likely originated from the coral host (23% of all transcripts in the quality-filtered metatranscriptome). In contrast to the holobiont-level profiles above, MDS ordination of host gene expression profiles revealed high similarity between healthy tissue samples (Fig. 2). Gene expression profiles of GA-affected and GA-unaffected samples on the other hand varied considerably, and were distinct from healthy samples. Additionally, gene expression profiles varied greatly among diseased colonies (i.e. colonies with GA), while gene expression profiles within each colony (i.e. between GA-affected and GA-unaffected tissues derived from the same colony) remained nearly identical in three out of six colonies. This finding suggests that GA has a variety of genetic effects at the colony level, including on seemingly healthy (GA-unaffected) tissue. Several factors may account for this effect, including the possibility that the sampled GA colonies may represent different stages in the progression of the disease or different coral genotypes. While samples were chosen from mature colonies with well-developed lesions, they may have come from morphologically similar, but pathologically different stages with underlying differences in gene expression patterns. Alternatively, the genetic background of the host or holobiont, which in turn may be correlated with disease status, may be driving the gene expression patterns we identified within and among diseased colonies.

**Fig. 3:**
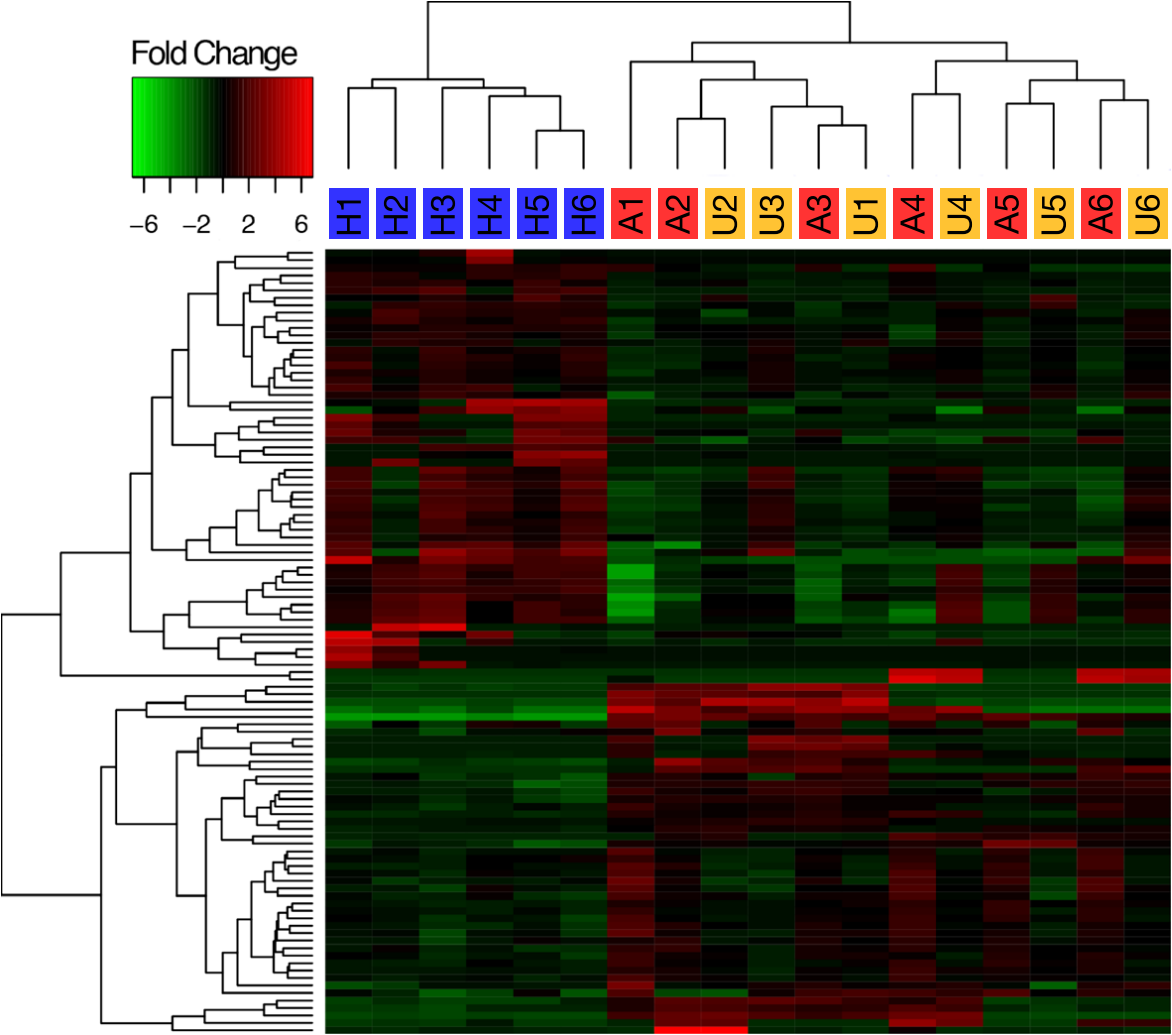
Heat map and clustering dendrograms of differentially expressed coral host genes among tissue types. Tissue samples are represented in columns, with labels designating H = healthy, A = GA-affected and U = GA-unaffected samples (label colors as in Fig. 2). Numbers designate the coral colony from which samples were collected (A and U samples with the same number were obtained from the same colony). Differentially expressed genes (DEG) are represented in rows, with heat map colors corresponding to log_2_ fold-change in FPKM values. Most DEGs are found between healthy and GA-diseased samples, both GA-affected and GA-unaffected

**Fig. 4:**
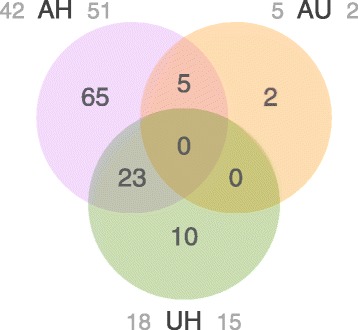
Venn diagram displaying the number and overlap of differentially expressed genes (DEGs). Labels designate comparisons between H = healthy, A = GA-affected, and U = GA-unaffected tissue. Gray numbers to the left and right of each label indicate the number of upregulated DEGs in this tissue type with respect to the other in each comparison

Coral gene expression profiles were also characterized by a notable number of DEGs among coral tissue types. In total, our pairwise DGE analyses revealed 105 unique, differentially expressed genes among the three tissue types (Fig. 3; see Additional file 2: Table S1 for list of genes, annotations and statistics). Of these, 93 genes were differentially expressed between healthy and GA-affected tissue types (Fig. 4) – more than any other pairwise comparison, and indicative of the significant impact that GA lesions have on coral physiology. The impact of GA on the entire coral colony was demonstrated by the fact that 33 genes were also differentially expressed between GA-unaffected (that is, seemingly healthy) and healthy tissue types. Interestingly, only seven genes were differentially expressed between GA-affected and GA-unaffected tissue, which is further evidence for the effect of GA on the entire colony.

Gene Ontology (GO) term and pathway analyses provide only limited insight into the molecular and biological role of these genes in GA. Only about half of the coral genes could be annotated with GO terms, and in most cases only a very small number of genes (≤ 3 each) was found in overrepresented, informative GO categories. These included the Wnt signaling pathway among genes upregulated in healthy tissue with respect to GA-affected tissue (GO:0016055, 3 genes, *p*-value <0.00005), and protein metabolic processes among genes upregulated in healthy tissue with respect to GA-unaffected tissue (GO:0019538, 3 genes, *p*-value <0.05). Pathway analyses using Uniprot accession identifiers of the DEGs did not indicate significant over-representation of genes by pathway. This lack of resolution was likely due to the small number of DEGs in each comparison, especially when analyzing up- and downregulated genes separately. Assigning few genes to GO terms is a common problem for studies of non-model organisms, particularly when those are distantly related to the model organisms from which GO annotations have been built.

### Genes involved morphogenesis and skeleton formation

While GO term enrichment and pathway analyses proved largely uninformative to characterize the transcriptomic changes associated with GA, their small number made it possible to evaluate gene function for all DEGs individually. Despite the limitations of translating gene function across distantly related organisms, we sought to elucidate the molecular and physiological processes affected in the coral host by combining information about DEGs from several sources, including homology to Uniprot entries and the annotated predicted proteome of *A. digitifera* (Additional file 2: Table S1). Unless noted otherwise, further support for putative gene functions was obtained by conserved domain analysis [28]. This manual approach suggested that GA is associated with substantial alterations of expression in genes involved in morphogenesis, organogenesis, and immune response.

Notably, we found multiple putative members of the Wnt signaling pathway among the DEGs, including homologs to vertebrate genes encoding Wnt proteins, low-density lipoprotein receptor-related proteins (LRPs), and a representative of the Frizzled receptor family (frizzled class receptor 5). Wnt signaling pathways are known to control body axis patterning and cell differentiation during embryonic development in all extant metazoans including Cnidaria (reviewed in [29]). Wnt signaling also plays a role in oncogenesis, and is essential for adult tissue regeneration in animals, an ability that is particularly well developed in Cnidaria (reviewed in [30]). Intriguingly, some LRPs are involved in the regulation of bone growth, and affect bone density, mass and development in vertebrates [31–33]. While we were able to confirm homology of two DEGs to genes encoding these vertebrate LRPs (*LRP4* and *LRP6*) by conserved domain analysis, LRP function in Cnidaria is poorly studied [30] and cannot be inferred from distantly related model organisms with any certainty. However, downregulation of *LRP* genes in GA-affected coral tissue along with reduced skeletal density in GA lesions [16] would be consistent with a role of LRPs in the formation of the skeleton in *M. capitata*, as well as previous studies of bone formation in other species. We thus believe this observation might provide a fruitful avenue for future research on the molecular underpinnings of GA.

Another ancient metazoan pathway regulating cell differentiation and development in Cnidaria is the Notch signaling pathway [34], which also seems affected by GA. Among DEGs associated with GA, we identified putative homologs of several mammalian genes involved in Notch signaling, including genes encoding Notch-regulated ankyrin repeat-containing protein, HES-1, and HES-4A. Though multiple coral proteins were annotated as HES-1 and -4A based on homology search, protein domain analysis reveals that the Hairy orange domain, which confers specificity of HES transcription factors, is not present in the coral protein, allowing us to conclude only that these genes are transcription factors. Gene expression of various transcription factors acting in a multitude of developmental contexts is presumably altered by GA as well: among others, homologs of *twist*, *sprouty 2*, as well as several helix-loop-helix, forkhead and homeodomain-containing genes (e.g. similar to *engrailed* and *muscle segment homeobox 3*) were found to be differentially expressed.

Significant changes in the expression of genes and pathways implicated in morphogenesis and cell differentiation may explain the gross tissue abnormalities in GA-diseased corals, and be linked to alterations in the deposition of the skeleton. This finding is also consistent with changes in the transcription of several collagens, since the structure and composition of the extracellular matrix mediates important processes during tissue growth and cell differentiation, including osteogenesis [35, 36]. Further corroborating this is another differentially expressed transcript, which shows weak homology to the vertebrate *Bone morphogenetic protein 1* (*BMP1*) and contains a CUB domain that is almost exclusively observed in extracellular and plasma membrane-associated proteins. While it remains uncertain whether this gene is a Cnidarian ortholog of *BMP1*, it may similarly be involved in the formation of the extracellular matrix. Through the processing of procollagens [37], BMP1 is implicated in rare diseases causing deformed bones and growth deficiency in mammals [38–41]. Evidenced by low expression in the developmental stages of larvae, followed by increased expression in calcifying coral polyps, a putative *BMP1* homolog has been hypothesized to also play a role in coral skeletogenesis [42].

### Genes involved in immune response and oncogenesis

Several genes similar to mammalian *tumor necrosis factor receptor-associated factors* (*TRAF*s) were discovered among genes upregulated in GA-affected tissue compared to healthy tissue (Additional file 2: Table S1). *TRAF* homologs have been described from a wide range of metazoans, including insects and hydroids [43, 44]. Tumor necrosis factor (TNF) ligands and receptors have been identified in the closely related coral *Acropora digitifera* in abundance higher than that found in humans, and have been shown to have conserved functions including apoptosis, caspase activation, coral bleaching and cell death [45]. Pathways connected to *TRAF*s, like Toll-like receptor signaling, are also evolutionarily ancient regulators of innate immunity and inflammation (reviewed in [46]). Upregulation of *TRAF* homologs in GA-tissue is therefore consistent with the activation of the immune system in diseased corals, likely in response to infection by opportunistic pathogens. This conclusion receives additional support by the upregulation of a Macrophage mannose receptor homolog, and the findings of Zhang et al. [11].

While many of the genes discussed above – including Wnt pathway genes and *TRAF*s – have been shown to be involved in tumor formation in other organisms (reviewed in [47, 48]), the low number of DEGs is not consistent with the systemic changes expected from extensive neoplasia. Likewise, neither cell cycle control, metabolism, nor DNA repair pathways seem to be strongly affected by GA, contrary to expectations raised by the hypothesis that GA lesions represent neoplastic tissue growth. Oncogenesis-related genes reported by Zhang et al. [11] to be associated with GA in *P. carnosa* were not found in *M. capitata*. Although one DEG was indicated as a possible *Deleted in malignant brain tumors 1* (*DMBT1*) homolog – a gene implicated in the immune response and epithelial cell differentiation [49], and linked to various human cancers [49–54] – the putative homolog was not supported by conserved domain analysis. The partial sequence homology according to BLAST-based approaches (as in Additional file 2: Table S1) indicated a match of a conserved SR domain, yet the transcript was found to be lacking the zona pellucida, CUB, and C-terminal transmembrane domains typical for *DMBT1* [28]. While parallels between GA and neoplasia cannot be ruled out, the transcriptomic signature of GA points to more limited effects connected to structural alterations of the coral tissue, especially the skeleton, and a state of inflammation/infection. Whether these are a cause or consequence of GA remains an open question for now.

### Growth anomaly and Symbiodinium clade

Since previous studies have identified at least two co-occurring *Symbiodinium* clades in our study population at Wai‘ōpae [22], we extracted three common taxonomic marker genes – *ITS-2*, *cp23S* and *psbA* – from the metatranscriptome assembly to determine the *Symbiodinium* clade composition in our colonies. All three genes consistently revealed that each colony harbors only one dominant clade, either C or D, with one exception (which contained both simultaneously). Further, both clades seem to be equally common in the population (C = 6, D = 5, C and D = 1). While *Symbiodinium* clade appears to have a large effect on gene expression at the holobiont level (Additional file 1: Figure S1), this is likely a result of insufficient ortholog detection. Since the *Symbiodinium* clades identified in our holobiont samples are only distantly related phylogenetically, even orthologous genes have diverged considerably between them, resulting in a misleading split of read counts between orthologous transcripts. As our current pipeline does not accurately account for this divergence, the relationship between GA and *Symbiodinium* gene expression requires an in-depth ortholog assignment and will be addressed in future research. Interestingly, *Symbiodinium* clade composition did seem to be correlated with coral host gene expression (Additional file 1: Figure S2). However, neither clade was significantly overrepresented in healthy or GA-affected colonies (Chi-square, *p* = 0.25), suggesting that neither clade C or D predispose the host towards developing GA. This is also supported by the fact that clade composition did not differ between affected and unaffected tissue in the same colony (with the exception of one colony housing mostly clade D in unaffected, but both clades C and D in affected tissue). Further investigation is underway to illuminate a possible link between *Symbiodinium* clade (in addition to the broader symbiont community) and GA.

## Conclusions

Extensive RNA-sequencing of healthy and GA-diseased tissue has provided a detailed molecular snapshot of gene expression in *M. capitata*, and represents the first transcriptome-scale resource for this important reef-building species in Hawai‘i. Through a combination of homology and GC content-based analyses we were able to identify a subset of transcripts that most likely originate from the coral host. Differential gene expression analysis among healthy, GA-affected and GA-unaffected coral tissue revealed gene expression patterns that are congruent with previous studies detailing the impact of GA on coral physiology, including immune system function and skeletal formation, but presumably not neoplastic tissue growth. This study represents one of the first to provide insight into the molecular etiology and pathology of this coral disease.

## Methods

### Sample collection

Collection of *M. capitata* fragments was carried out in January and February 2013 at Waiʻōpae, East Hawaiʻi Island (19°29′55′′ N, 154°49′06′′ W), a site known for high prevalence of GA in *M. capitata* [15]. Additional samples collected at Kīholo, West Hawaiʻi Island (19°51′9″ N, 155°55′55″ W) were included in the metatranscriptome assembly, but not considered in the DGE analysis due to the low sample size for that site. Healthy (*N* = 9) and GA-diseased (*N* = 9) coral colonies in 2–4 m depth were selected, and small fragments of approximately 1cm^3^ separated from the colony using a hammer and chisel. Sample collection was authorized by Hawaiʻi State Division of Aquatic Resources (Special Activity Permit 2013–33). One fragment was collected from each healthy colony (“healthy tissue”; *N* = 9), while pairs of fragments were collected from each colony with GA: GA lesion tissue (“GA-affected tissue”; *N* = 9) and apparently healthy tissue from the same colony (“GA-unaffected tissue”; *N* = 9). A total of 27 samples were collected from Waiʻōpae (six of each tissue type) and Kīholo (three of each tissue type). The coral fragments were immediately placed in liquid nitrogen for transport to the laboratory facility. Upon arrival at the laboratory, tissue from the coral fragments was scraped off using a sterile razor blade and crushed to a powder with mortar and pestle, using liquid nitrogen to prevent the samples from thawing during processing, and stored at −80 °C until RNA isolation.

### RNA extraction and sequencing

Total RNA was extracted from ~0.1 g tissue powder using a combination of TRIzol/ chloroform extraction and the RNeasy Mini Kit (Qiagen). First, samples were incubated for 5 min in TRIzol (Life Technologies; 1 ml per 0.1 g tissue) at room temperature, followed by centrifugation at 12,000×g at 4 °C for 10 min. After adding chloroform (Sigma-Aldrich; 0.2 ml per 1 ml TRIzol) to the supernatant, samples were mixed vigorously, incubated at room temperature for 3 min, and centrifuged at 18,000×g at 4 °C for 18 min. The aqueous phase was purified using an equal volume of 100% molecular grade ethanol (Sigma-Aldrich) and the RNeasy Mini Kit according to the manufacturer’s instructions. DNA was removed by implementing a 25 min DNA digestion step (Qiagen RNase-free DNase Set). RNA quality and quantity was determined using the Qubit RNA Broad Range Assay Kit (Life Technologies) and Agilent RNA 6000 Nano Kit (Agilent), and RNA aliquots were stored at −80 °C.

Once total RNA of sufficient quantity and quality was attained, 3 μg total RNA was sent to the Yale Center for Genomic Analysis (YCGA) for mRNA isolation and sequencing on three lanes of an Illumina HiSeq 2000. Strand-specific libraries were constructed using a modified protocol developed by YCGA (see Additional file 1: Methods S1). Paired-end sequencing was conducted for 75 cycles for each read pair, with samples multiplexed using the TruSeq Paired-End Cluster Kit v3-cBot-HS (Illumina).

### Metatranscriptome assembly and quality analysis

Sequencing generated a total of 687 million reads (103 gigabases), or 25 million reads on average per sample (range: 17–42 million paired-end reads). Raw data sequence quality was assessed using FastQC 0.11.4 [55] and quality filtered with Trimmomatic 0.32 [56], retaining 97.8% of read pairs. Data were then in silico normalized to 50× coverage using the normalization script provided with the Trinity package [57]. The normalized reads were assembled into a metatranscriptome using default Trinity parameters [58]. The metatranscriptome, consisting of 660,340 isotigs (“transcripts”) representing 441,520 unigenes (Table 1), was independently subjected to multiple filters as follows: Putative protein coding regions based on open reading frames were identified using the Trinity TransDecoder plugin [61], and transcripts with no ORF were discarded due to the increased difficulty in annotating non-coding RNAs for non-model organisms. Highly similar protein sequences were identified using CD-HIT with a similarity cutoff value of 0.9 [59]. For sequences with similarity greater than 90%, the longest sequence was retained as the representative sequence for that group. Low expression protein-coding transcripts were filtered on the basis of fragments per kilobase of transcript per million mapped reads (FPKM) values, calculated by RSEM [60], using a pooled sample cutoff value of 0.5. Sequences that met all the criteria of these filtering steps (87,085 transcripts representing 76,063 genes) were retained for further analysis, including host-symbiont separation.

### Coral transcriptome identification

In order to assess differential gene expression of the coral host, we developed the following methodology to identify transcripts of putative coral and non-coral (symbiont) origin. Coral host transcripts were identified from the quality-filtered metatranscriptome assembly using three methods independently: (1) detection of orthologous coral proteins based on reciprocal best hits to reference coral proteomes using InParanoid version 4.1 [62], (2) taxonomic annotation of cnidarian proteins based on BLASTp searches, and (3) use of a GC% content cutoff based on observed taxon-specific GC biases using Blobology [63]. Sequences that were classified as cnidarian based on homology search methods were classified as coral sequences for the purpose of this study, and included in the coral transcriptome subset. Similarly, symbiont transcripts were identified through the aforementioned three methods, and sequences that were classified as potential symbiont taxa were excluded from the coral transcriptome. GC% content cutoff was used as a final step to include sequences with no taxonomic annotation (coral or non-coral) that have a GC content signature similar to annotated cnidarian sequences.

Ortholog detection of coral and *Symbiodinium* proteins based on reciprocal best hits match to the predicted proteome of references was performed using InParanoid [62]. The coral references used for this analysis included the predicted proteomes based on the *Acropora digitifera* genome [64] and two transcriptomes produced by the Matz lab for *A. hyacinthus* and *A. tenuis* [65]. The predicted proteome based on the *Symbiodinium minutum* clade B1 genome was used as a *Symbiodinium* spp. reference [66] to identify potential symbiont proteins, and was also used as an outgroup in all *Acropora* ortholog analyses. Sequences identified as orthologous to *Acropora* proteins based on this analysis were classified as coral unless the sequence was also identified as symbiont-derived through homology search methods. Sequences identified as orthologous to *Symbiodinium* proteins based on InParanoid analysis were classified as non-coral.

Homolog detection of coral (BLAST hit to cnidarian taxa) and non-coral (BLAST hit to symbiont taxa) transcripts was determined using BLASTp. Sequences with BLASTp hits (e-value <1e–10) to Cnidaria (1564), Dinophyceae (2425), Bacteria (669) and Fungi (355) sequences in the National Center for Biotechnology Information (NCBI) non-redundant database, representing coral host and symbiont lineages, were identified. Sequences that were identified as homologous to cnidarian proteins were classified as coral unless the sequence was also identified as a symbiont protein based on InParanoid or BLASTp analyses. Sequences identified as homologous to coral symbionts were classified as non-coral.

Based on InParanoid and BLASTp analyses, we were only able to classify approximately 10% of the quality-filtered assembly as coral or non-coral. Definitive identification of coral transcripts based on orthology or homology is difficult due to a lack of substantial coral genetic data, as compared to model organisms. In order to include the maximum the number of potential coral transcripts in our analyses, we used taxon-annotated GC content analysis to determine a GC content cut-off to classify the remaining unannotated sequences as either coral or non-coral. We determined the relative GC content for each sequence in the quality-filtered assembly using Blobology [63], and found two major peaks of GC content-associated transcript abundance (Fig. 1). By overlaying GC content plots of annotated (coral and non-coral) sequences and the quality-filtered assembly, we found that the two peaks in GC-associated transcript abundance strongly correspond to coral host and non-coral sequences, respectively (Fig. 1). We therefore classified unannotated sequences from the quality-filtered assembly with GC content <47% as coral. As with previous coral transcript filtering steps, sequences with GC <47% were not classified as coral if they were identified as non-coral through homology analyses. These filtering steps resulted in a coral host transcriptome totaling 20,461 transcripts from 13,643 genes.

### Gene expression analyses

Raw reads of the Waiʻōpae samples were mapped to the filtered metatranscriptome assembly and coral transcriptome subset using RSEM [61] and bowtie 2.2.4 [67] to obtain read counts. Overall similarity of gene expression profiles were visualized by MDS ordination of filtered (minimum 1 count per million reads in at least two libraries) and normalized (to raw library size) counts using the default plotMDS function in edgeR 3.10.5 [68]. Differential gene expression was modeled for all pairwise comparisons of tissue types (healthy, GA-affected and GA-unaffected) using edgeR [68]. Differences in expression values with a FDR-adjusted *p*-value ≤0.01 and fold-change ≥2 were deemed significant. Both MDS and differential gene expression analyses were performed separately for the holobiont and coral host, using the quality-filtered metatranscriptome, and the putative coral transcriptome, respectively.

### Symbiodinium clade determination

To identify *Symbiodinium* lineages associated with *M. capitata*, and quantify their relative abundance in each colony, transcripts of three target genes were retrieved from the metatranscriptome assembly: nuclear rRNA, including the internal transcribed spacer *ITS-2*, chloroplast 23S rRNA (*cp23S*), and photosystem II protein D1 (*psbA*). For each gene, reference sequences of clades A–H [69] obtained from NCBI GenBank were used as blastn queries, and aligned with MAFFT 7 [70] to hits exceeding an e-value cutoff of e^−10^ and a length cutoff of 50%. Phylogenetic analyses were performed to establish which lineage each transcript represents, using the neighbor joining method (Jukes-Cantor model) implemented in MAFFT. Expression levels of each transcript measured in FPKM values were then compared to assess the *Symbiodinium* community composition in each coral sample.

### Gene annotation

Transcript identity and putative function were assessed using four approaches: (1) BLASTp 2.3.0 searches against the National Center for Biotechnology Information non-redundant and Uniprot peptide databases (an e-value cutoff of 1e^−4^ was applied in both cases), (2) HMMER v3.1b2 [59] searches against the Pfam-A database, (3) the online KEGG GhostKOALA search program [71], and (4) online ZoophyteBase search program [72]. Blast-based annotations were evaluated for subsets of genes and their putative homologs by assessing concordance of conserved domains identified through the NCBI conserved domain database [28]. Gene Ontology terms for coral transcripts were acquired by using mapping files available from the Gene Ontology Consortium [73] to compile GO terms associated with Pfam, Uniprot and KEGG annotations, and by running InterProScan as implemented in Blast2GO [74] under default parameters. Differentially expressed genes were tested for overrepresentation of biological process GO terms with the R package GOstats v1.7.4 using a hypergeometric test with a *p*-value cut-off of 0.05. Protein searches against the *A. digitifera* predicted proteome [72] were used to extract putative coral ortholog annotations from the KEGG orthology-annotated database. An additional file is available with annotations from multiple sources along with DEG statistics (Additional file 2: Table S1).

## Additional files

Additional file 1: **Figure S1.** Shows two MDS plots of holobiont gene expression profiles compared across tissue types and *Symbiodinium* clade harbored by coral host. **Figure S2.** A MDS plot of coral host gene expression compared across Symbiodinium clade harbored by coral host. **Methods S1.** Includes library preparation and data processing, and commands used in bioinformatics analyses. (PDF 179 kb)

Additional file 2: Table S1. Shows differentially expressed coral genes along with results from pairwise tissue type comparisons, gene annotations, annotation sources, and gene expression statistics. (XLS 172 kb)

## Abbreviations

*BMP1*: Bone morphogenetic protein 1
DEG: Differentially expressed gene
DGE: Differential gene expression
*DMBT1*: Deleted in malignant brain tumors 1
FDR: False discovery rate
FPKM: Fragments per kilobase of transcript per million mapped reads
GA: Growth anomaly
GO: Gene ontology
*LRP*: Low-density lipoprotein receptor-related protein
MDS: Multidimensional scaling
NCBI: National Center for Biotechnology Information
ORF: Open reading frame
TNF: Tumor necrosis factor
*TRAF*: Tumor necrosis factor receptor-associated factor
YCGA: Yale Center for Genomic Analysis
