# Supplementary material for: De novo metatranscriptome assembly and coral gene expression profile of Montipora capitata with growth anomaly
Source: BMC Genomics. 2017 Sep 11;18:710. doi: 10.1186/s12864-017-4090-y (PMC5594617; doi:10.1186/s12864-017-4090-y)
Supplement: Supplementary file 1 — Figure S1. Shows two MDS plots of holobiont gene expression profiles compared across tissue types and Symbiodinium clade harbored by coral host. Figure S2. A MDS plot of coral host gene expression compared across Symbiodinium clade harbored by coral host. Methods S1. Includes library preparation and data processing, and commands used in bioinformatics analyses. (PDF 179 kb) [file 12864_2017_4090_MOESM1_ESM.pdf]

Additional File 1

De novo metatranscriptome assembly and coral gene expression profile of _Montipora capitata_ with growth anomaly

Monika Frazier, Martin Helmkampf, M. Renee Bellinger, Scott Geib, Misaki Takabayashi

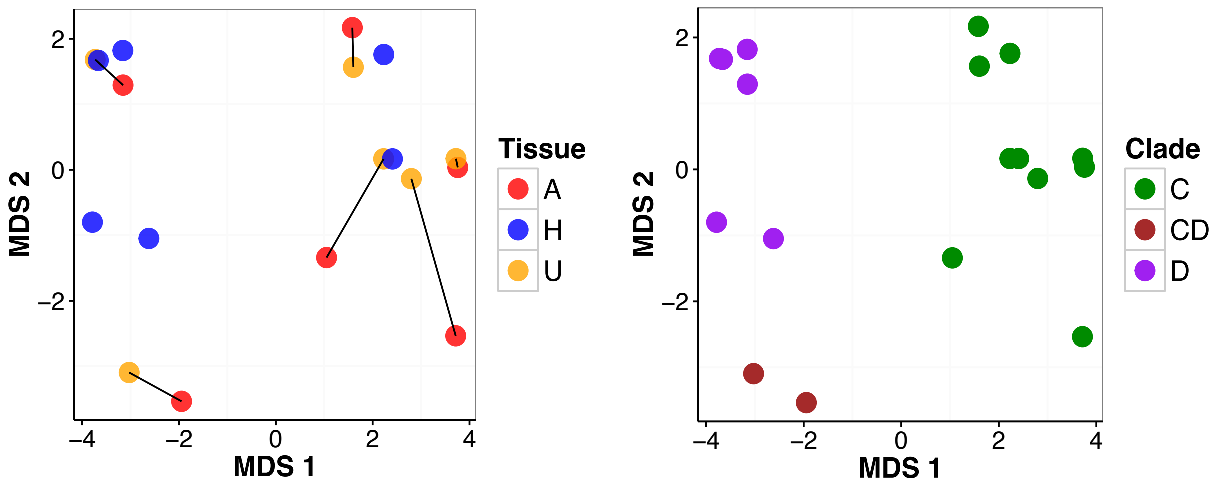

Figure S1. Holobiont gene expression profiles. Samples are colored according to tissue type (A=GA-affected, U=GA-unaffected, H=healthy tissue) and dominant *Symbiodinum* clade, and were mapped by metric multidimensional scaling (MDS). Distances between each pair of samples represents the typical $\log_2$ fold-change in gene expression between transcripts. Black lines connect GA-affected and GA-unaffected samples obtained from the same colony.

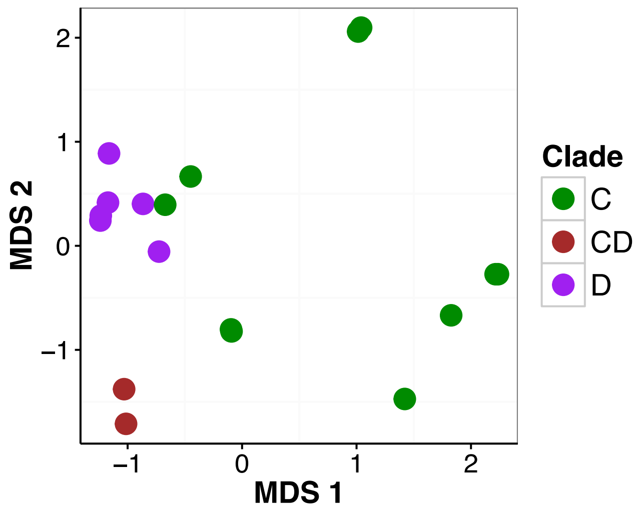

Figure S2. Coral host gene expression profiles. Samples are colored according to dominant *Symbiodinum* clade, and were mapped by metric multidimensional scaling (MDS). Distances between each pair of samples represent the typical $\log_2$ fold-change in gene expression between coral host transcripts.

Methods S1

Library Preparation Protocol (Yale Center for Genomic Analysis)

RNA Seq Quality Control: Total RNA quality is determined by estimating the A260/A280 and A260/A230 ratios by nanodrop. RNA integrity is determined by running an Agilent Bioanalyzer gel, which measures the ratio of the ribosomal peaks.

RNA Seq Library Prep:mRNA is purified from approximately 500ng of total RNA with oligo-dT beads and sheared by incubation at 94C.Following first-strand synthesis with random primers, second strand synthesis is performed with dUTP for generating strand-specific sequencing libraries.The cDNA library is then end-repaired, and A-tailed, adapters are ligated and second-strand digestion is performed by Uricil-DNA-Glycosylase. Indexed libraries that meet appropriate cut-offs for both are quantified by qRT-PCR using a commercially available kit (KAPA Biosystems) and insert size distribution determined with the LabChip GX. Samples with a yield of ≥0.5 ng/ul are used for sequencing.

Flow Cell Preparation and Sequencing: Sample concentrations are normalized to 2 nM and loaded onto Illumina version 3 flow cells at a concentration that yields 170-200 million passing filter clusters per lane. Samples are sequenced using 75 bp paired end sequencing on an Illumina HiSeq 2000 according to Illumina protocols. The 6 bp index is read during an additional sequencing read that automatically follows the completion of read 1.Data generated during sequencing runs are simultaneously transferred to the YCGA high performance computing cluster. A positive control (prepared bacteriophage Phi X library) provided by Illumina is spiked into every lane at a concentration of 0.3% to monitor sequencing quality in real time.

Data Analysis and Storage: Signal intensities are converted to individual base calls during a run using the system's Real Time Analysis (RTA) software.Base calls are transferred from the machine's dedicated personal computer to the Yale High Performance Computing cluster via a 1 Gigabit network mount for downstream analysis.Primary analysis - sample de-multiplexing and alignment to the human genome - is performed using Illumina's CASAVA 1.8.2 software suite. The data are returned to the user if the sample error rate is less than 2% and the distribution of reads per sample in a lane is within reasonable tolerance. Data is retained on the cluster for at least 6 months, after which it is transferred to a tape backup system.

Command line scripts for bioinformatic analyses

FastQC: Assess Raw Reads Quality

for FILE in *Sample1 Sample2 Sample3*

do ./FastQC/fastqc -t 32 $FILE.fastq

done

Trimmomatic: Trim Raw Data

Parameters:

IN1=A1.R1.fastq

IN2=A1.R2.fastq

WINSIZE=5

WINCUTOFF=20

LEADING=20

TRAILING=20

MINLEN=50

Command used:

java -jar trimmomatic-0.32.jar PE -phred33 $IN1 $IN2

trimmed.$IN1.$WINSIZE.$WINCUTOFF.R1.fastq

single.$IN1.$WINSIZE.$WINCUTOFF.R1.fastq

trimmed.$IN2.$WINSIZE.$WINCUTOFF.R2.fastq

single.$IN2.$WINSIZE.$WINCUTOFF

.R2.fastq ILLUMINACLIP:TruSeq3-PE.fa:2:30:10 LEADING:$LEADING

TRAILING:$TRAILING SLIDINGWINDOW:$WINSIZE:$WINCUTOFF

MINLEN:$MINLEN

Trinity: Assemble Transcriptome

Required programs in path:

bowtie

samtools

Perl modules: PerlIO::gzip.pm

Command used:

/path/Trinity --seqType fq --JM 200G --left all.trimmed.R1.fastq --right all.trimmed.R2.fastq --SS_lib_type RF --CPU 32 --normalize_reads

TransDecoder: Translate Amino Acid

Required programs/files in path:

PfamA.hmm

cd-hit

hmmer

Command used:

/path/trinity-plugins/TransDecoder_r20131110/TransDecoder -t Trinity.fasta --reuse -S --search_pfam */path*/Pfam-A.hmm --MPI --CPU 100 --cd_hit_est */path*/cd-hit-est

CD-HIT: Cluster Similar Proteins

Command used:

/path/cd-hit -i Trinity.fasta.transdecoder.pep -M 0 -T 0 -g 1 -c 0.5 -n 2 -o *Output_File*

BLAST: Protein Annotation

Commands used:

/path/blastp -query *Input_file* -db nr -out *Output_file* -evalue 1e-4 -num_threads 50 -max_target_seqs 1 -outfmt 6

/path/blastp -query *Input_file* -db uniprot_sprot.fasta -out *Output_file* -evalue 1e-4 -num_threads 50 -max_target_seqs 1 -outfmt 6

RSEM: Align raw reads to assembly

Required programs in path:

bowtie

samtools

RSEM

Command used:

/path/trinityrnaseq_r20140717/util/align_and_estimate_abundance.pl --transcripts Trinity.fasta --left all.trimmed.R1.fastq --right all.trimmed.R2.fastq --seqType fq --SS_lib_type RF --thread_count 32 --est_method RSEM --aln_method bowtie2 --trinity_mode --prep_reference

Parse results file:

FPKM=*value*

cat RSEM.genes.results | sed '1,1d' | awk '$7 >= $FPKM' | wc –l

Inparanoid: Identify Acropora and Symbiodinium inparalogs

Required programs in path:

blastall

formatdb

Command used:

perl inparanoid.pl *Assembled_transcriptome Acropora_reference Symbiodinium_reference*

RSEM: Align raw reads and estimate gene expression

Required programs in path:

bowtie

samtools

RSEM

Command used:

/*path*/ trinityrnaseq_r20140717/util/align_and_estimate_abundance.pl --transcripts *Transcriptome_assembly* --left *Sample_name*.R1.fastq --right *Sample_name*.R2.fastq --seqType fq --SS_lib_type RF --thread_count 32 --est_method RSEM --aln_method bowtie2 --output_prefix *Sample_name*--trinity_mode --prep_reference

EdgeR: Determine differentially expressed genes

Required programs in path:

R

Command used:

/*path*/trinityrnaseq_r20140717/util/abundance_estimates_to_matrix.pl --est_method RSEM *Sample1*.genes.results *Sample2*.genes.results –out_prefix *Output_file*
